# P-519. Detection & Severity of Respiratory Virus Co-Infections with SARS-CoV-2, Influenza, or RSV in U.S. Children <2 Years Old

**DOI:** 10.1093/ofid/ofaf695.734

**Published:** 2026-01-11

**Authors:** Annette Regan, Stacey Rowe, Onyebuchi Arah, Flor M Munoz, Eileen Fry-Bowers, Sheena G Sullivan

**Affiliations:** Kaiser Permanente Southern California, Pasadena, CA; Kaiser Permanente Southern California, Pasadena, CA; University of California Los Angeles, Los Angeles, California; Baylor College of Medicine Houston, Dallas, Texas; University of San Francisco, San Francisco, California; Monash University, Melbourne, Victoria, Australia

## Abstract

**Background:**

Co-infections with multiple respiratory viruses may increase the severity of illness in young children, introducing a greater healthcare burden compared to single respiratory virus infections.

**Figure:**
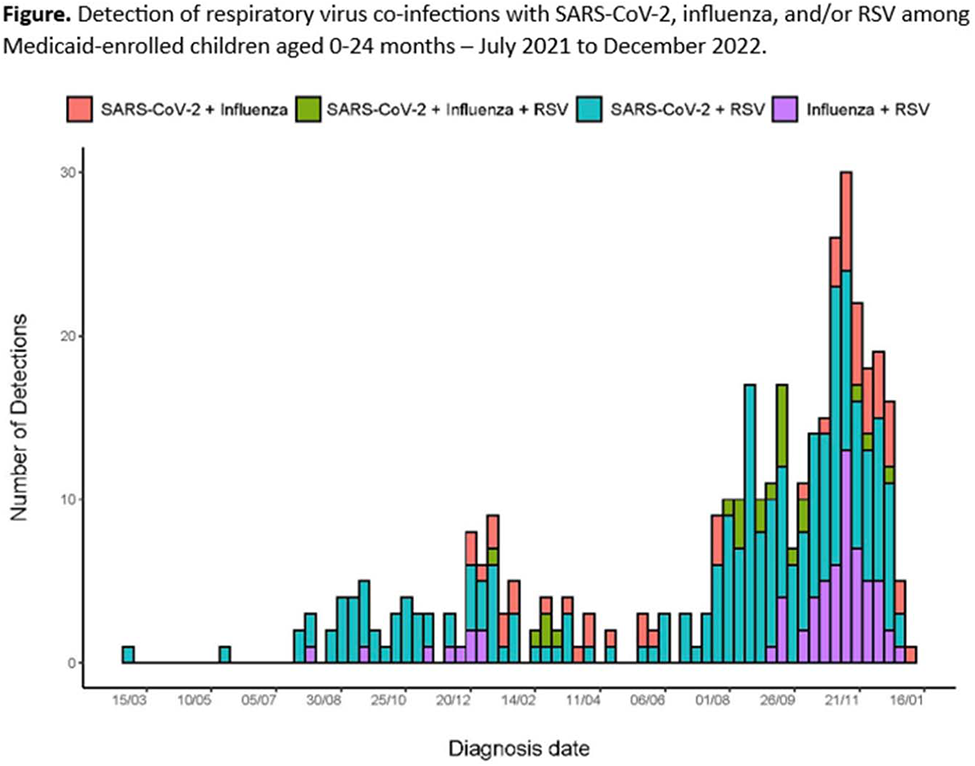

**Figure:**
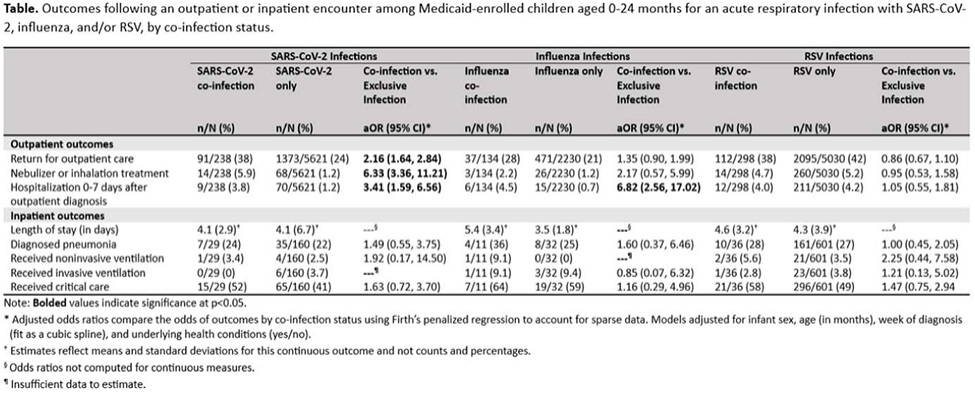

**Methods:**

We conducted a national cohort study of Medicaid-enrolled children aged 0-24 months presenting for outpatient or inpatient care and tested by multiplex RT-PCR for SARS-CoV-2, influenza, and RSV between 1 July 2021 and 31 December 2022 using the Merative® MarketScan Multi-State Medicaid Database. ICD-10-CM and procedure codes were used to identify clinical diagnoses and the date of clinical testing. We compared the odds of outpatient encounters returning for additional outpatient care, receiving nebulizer treatment, and requiring hospitalization and the odds of inpatient encounters developing pneumonia, receiving noninvasive/invasive ventilation, or requiring critical care by co-infection status using Firth’s penalized logistic regression. Adjusted odds ratios (aOR) controlled for infant sex, age, week of diagnosis, and underlying health conditions.

**Results:**

Among 40,527 outpatient encounters, 13,230 (33%) were positive for a respiratory virus: 5,621 (14%) for SARS-CoV-2 only, 5,030 (12%) for RSV only, 2,230 (5.5%) for influenza only, and 349 (0.9%) for multiple viruses. Among 954 inpatient encounters, 310 (32%) were positive for a respiratory virus: 211 (22%) for RSV only, 70 (7.3%) for SARS-CoV-2 only, 15 (1.6%) for influenza only, and 14 (1.5%) for multiple viruses. The most common co-infection was SARS-CoV-2 and RSV for outpatient (n=215/349; 62%) and inpatient co-infections (n=8/14; 57%)(Figure). The odds of returning to outpatient care (aOR: 2.16; 95%CI 1.64, 2.84), receiving nebulizer treatment (aOR: 6.33; 95%CI 3.36, 11.21), and hospitalization (aOR: 3.41; 95%CI 1.59, 6.56) were higher among SARS-CoV-2 co-infections compared to SARS-CoV-2 only (Table). In-hospital outcomes did not differ by co-infection status.

**Conclusion:**

Children with an outpatient-attended SARS-CoV-2 or influenza co-infection, which were mostly attributed to RSV, more frequently required hospitalization and additional medical care. Given the burden of RSV and co-infections, RSV prevention remains a priority and a possibility through maternal vaccine and monoclonal antibodies.

**Disclosures:**

Annette Regan, PhD, MPH, Moderna: DSMB Membership Stacey Rowe, PhD, MPH, CSL Seqirus: Advisor/Consultant Flor M. Munoz, MD, Merck: Advisor/Consultant|Pfizer: Advisor/Consultant|Pfizer: Grant/Research Support Sheena G. Sullivan, PhD, Astra-Zeneca: Advisor/Consultant|CSL Behring and CSL Seqirus: Advisor/Consultant|GSK: Advisor/Consultant|Moderna: Advisor/Consultant|Novavax: Advisor/Consultant|Pfizer: Advisor/Consultant|Sanofi: Advisor/Consultant

